# State-Specific Patterns of Cigarette Smoking, Smokeless Tobacco Use, and E-Cigarette Use Among Adults — United States, 2016

**DOI:** 10.5888/pcd16.180362

**Published:** 2019-02-07

**Authors:** S. Sean Hu, David M. Homa, Teresa Wang, Yessica Gomez, Kimp Walton, Hua Lu, Linda Neff

**Affiliations:** 1 Office on Smoking and Health, National Center for Chronic Disease Prevention and Health Promotion, Centers for Disease Control and Prevention, Atlanta, Georgia; 2 Division of Population Health, National Center for Chronic Disease Prevention and Health Promotion, Centers for Disease Control and Prevention, Atlanta, Georgia

## Abstract

**Introduction:**

State-level monitoring of changes in tobacco product use can help inform tobacco control policy and practice. This study examined state-specific prevalence of cigarette, smokeless tobacco, and e-cigarette use among US adults.

**Methods:**

Data came from the 2016 Behavioral Risk Factor Surveillance System (BRFSS), a state-based telephone survey of US adults aged 18 years or older (N = 477,665). Prevalence estimates for current (every day or some days) cigarette smoking, smokeless tobacco use, and e-cigarette use were calculated for all 50 states and the District of Columbia (DC) and stratified by sex and race/ethnicity. Because the 2016 BRFSS measured e-cigarette use for the first time, estimates of ever e-cigarette use and concurrent use of cigarettes and e-cigarettes were also calculated. We assessed subgroup differences with χ^2^ tests.

**Results:**

In 2016, prevalence of current cigarette smoking among US adults ranged from 8.8% (Utah) to 24.8% (West Virginia), while prevalence of current smokeless tobacco use ranged from 1.3% (DC) to 9.8% (Wyoming). For e-cigarettes, ever use ranged from 16.2% (DC) to 28.4% (Arkansas), and current use ranged from 2.4% (DC) to 6.7% (Oklahoma). Across all states, current e-cigarette use was significantly higher among current cigarette smokers than among former or never cigarette smokers. States with the highest prevalence of cigarette smoking generally had a high prevalence of current e-cigarette use.

**Conclusion:**

Prevalence of adult cigarette smoking, smokeless tobacco use, and e-cigarette use varies across states. These findings underscore the importance of comprehensive statewide tobacco control and use prevention efforts that address the diverse tobacco products used among adults.

**Summary**

**What is already known on this topic?**

Cigarettes and smokeless tobacco are the most prevalent forms of tobacco used among adults. Furthermore, the use of emerging tobacco products such as e-cigarettes has increased among adults, particularly among current and former adult cigarette smokers.

**What is added by this report?**

In US states in 2016, as many as 1 in 4 adults were current cigarette smokers (West Virginia); 1 in 10 adults currently used smokeless tobacco products (Wyoming); and 1 in 15 adults currently used e-cigarettes (Oklahoma).

**What are the implications for public health practice?**

Continued implementation of proven population-based interventions can help reduce adult tobacco use across tobacco product types, particularly in states with the highest prevalence of use.

## Introduction

Tobacco product use is the leading cause of preventable disease and death in the United States. Cigarette smoking, in particular, harms nearly every organ in the body and is responsible for about 480,000 US deaths annually (1). Despite progress in reducing cigarette smoking in the United States, the tobacco product landscape has diversified. In 2015, about 1 in 5 US adults currently used some form of tobacco product (2). Cigarettes and smokeless tobacco are the most prevalent forms of tobacco used among adults (2–4). Although the prevalence of adult cigarette smoking has declined in many states, there has been little change in the prevalence of current smokeless tobacco use among adults in most states, and in some states the use of these products has increased (3,5). Furthermore, the use of emerging tobacco products such as e-cigarettes has increased among adults, particularly among current and former adult cigarette smokers (6,7).

Many US adults are also using multiple tobacco products (2). Although different tobacco products can have varying levels of addictive potential and harmful substances, concurrent use of multiple tobacco products can increase exposure to harmful and carcinogenic chemicals (1,8). The prevalence of adults who report concurrent use of cigarettes and smokeless tobacco varies across states, and national data indicate that nearly 60% of current adult e-cigarette users were also current cigarette smokers in 2015 (2,3,9–11). Smokeless tobacco and e-cigarettes are marketed as alternatives to cigarette smoking (6,12). During 2014–2016, of US smokers who made a quit attempt within the past year, 35.3% reported using e-cigarettes during their quit attempts (13). E-cigarettes may help nonpregnant adult smokers if used as a complete substitute for all cigarettes and other smoked tobacco products; however, e-cigarettes are not approved by the Food and Drug Administration as a quit-smoking aid, and the US Preventive Services Task Force concluded that evidence is insufficient to recommend e-cigarettes for smoking cessation in adults, including pregnant women (6,13,14).

Although previous studies have examined the state-specific prevalence of adult cigarette and smokeless tobacco use and concurrent use of these products (3–5), only 1 study to date has assessed state prevalence of adult e-cigarette use by using data from the 2014–2015 Tobacco Use Supplement to the Current Population Survey (10). However, that study did not assess differences by sociodemographic characteristics such as sex and race/ethnicity. More recent estimates of state-specific e-cigarette use among adults, including by demographics and cigarette smoking status, have not been reported. Therefore, the objective of this study was to report state-specific estimates of cigarette smoking, smokeless tobacco use, and e-cigarette use, as well as concurrent cigarette smoking and e-cigarette use, among US adults by using the 2016 Behavioral Risk Factor Surveillance System (BRFSS).

## Methods

### Data source

The BRFSS survey is an annual state-based random-digit–dialed telephone (landline and cellular telephone) survey, which has tracked health conditions and risk behaviors throughout the US annually since 1984. With support from the Centers for Disease Control and Prevention (CDC), the BRFSS survey is conducted by all 50 state health departments as well as those in the District of Columbia (DC), Puerto Rico, Guam, and the US Virgin Islands. Every month, data on health-related risk behaviors, clinical preventive health practices, and access to and use of health-care services are collected from a randomly selected, representative sample of noninstitutionalized adults aged 18 years or older residing in the United States. During 2016, a total of 477,665 respondents from all 50 states and DC completed the survey; the median survey response rate for all states, territories, and DC was 47.0% (range, 30.7%–65.0%). Detailed information about the BRFSS survey design, methods, and questionnaire are available at www.cdc.gov/brfss.

### Measures

Three tobacco product types were assessed in the 2016 BRFSS Core Section: cigarettes, smokeless tobacco products (including chewing tobacco, snuff, and snus), and e-cigarettes. Current cigarette smokers were persons who reported smoking at least 100 cigarettes in their lifetime and smoking every day or some days at the time of the survey. Former cigarette smokers were persons who reported smoking at least 100 cigarettes in their lifetime but smoking “not at all” at the time of the survey. Never cigarette smokers were persons who never smoked or who smoked less than 100 cigarettes in their lifetime. Current smokeless tobacco use was defined as using chewing tobacco, snuff, or snus every day or some days at the time of the survey. Current e-cigarette use was defined as using e-cigarettes or other electronic “vaping” products every day or some days at the time of survey. Ever e-cigarette use was defined as having ever using an e-cigarette or other electronic “vaping” product, even just 1 time, during the respondent’s lifetime.

### Analysis

Prevalence estimates with 95% confidence intervals were calculated for current cigarette smoking, current smokeless tobacco use, and current and ever e-cigarette use. Estimates were calculated overall, by state, and by sex. Data were additionally stratified by race/ethnicity for ever e-cigarette use. Because of the limited sample sizes, these stratifications were not performed for current cigarette smoking, smokeless tobacco use, or e-cigarette use. Race/ethnicity groups were categorized as non-Hispanic white, non-Hispanic black, Hispanic, and non-Hispanic other (Asian, Native Hawaiian or other Pacific Islander, American Indian or Alaska Native, or some other group). The prevalence of current, former, and never cigarette smokers among current e-cigarette users was also assessed. Estimates for current cigarette smoking and current e-cigarette use were stratified into quartiles and mapped to illustrate the relative distribution of prevalence by state.

Data were weighted to yield state representative estimates while taking into account the probability of selection and adjusting for nonresponse bias and noncoverage errors. We used χ^2^ tests to assess differences among groups, with *P* < .05 considered to be significant. All analyses were conducted using SAS version 9.4 (SAS Institute, Inc) survey procedures to account for the complex survey sampling design.

## Results

### Cigarettes

Prevalence of current cigarette smoking ranged from 8.8% (Utah) to 24.8% (West Virginia) (Table 1). By sex, the prevalence of cigarette smoking was significantly higher among men than women in 40 states and DC. Estimates ranged from 10.4% (Utah) to 25.8% (West Virginia) among men and from 7.1% (Utah) to 24.0% (Kentucky) among women. Seven of the 10 states with the highest prevalence of current cigarette smoking were in the South (West Virginia [24.8], Kentucky [24.5], Arkansas [23.6], Louisiana [22.8], Mississippi [22.7], Tennessee [22.1], and Alabama [21.5]), while the remaining 3 states were in the Midwest (Ohio [22.5], Missouri [22.1], and Indiana [21.1]). In contrast, 8 of the 10 states with the lowest prevalence of current cigarette smoking were in the West (Utah [8.8], California [11.0], Hawaii [13.1], and Washington [13.9]) or Northeast (Connecticut [13.3], Massachusetts [13.6], New Jersey [14.0], and New York [14.2]); the remaining 2 states were in the South (Maryland [13.7] and Texas [14.3]).

**Table 1:** State-Specific Prevalence of Current Cigarette Smoking^a^, Current Smokeless Tobacco Use^b^, and Current E-cigarette Use^c^ Among Adults Aged 18 Years or Older, by Sex — Behavioral Risk Factor Surveillance System, United States, 2016

State (Sample Size)	Cigarette Smoking, % (95% CI)			Smokeless Tobacco Use, % (95% CI)			E-cigarette Use, % (95% CI)		
	Total	Men	Women	Total	Men	Women	Total	Men	Women
All 50 states and District of Columbia median (N = 477,665)	17.1 (15.6–18.5)	18.8 (16.0–21.6)	15.3 (13.7–16.9)	4.0 (3.3–4.7)	7.2 (5.9–8.5)	1.0 (0.5–1.4)	4.7 (3.9–5.5)	5.6 (4.8–6.4)	3.8 (2.6–5.1)
Alabama (n = 7,031)	21.5 (20.2–22.9)	23.3 (21.1–25.5)	20.0 (18.2–21.7)	5.6 (4.8–6.4)	10.5 (8.9–12.1)	1.2 (0.8–1.6)	5.1 (4.3–5.9)	6.5 (5.1–7.9)	3.9 (3.1–4.7)
Alaska (n = 2,914)	19.0 (16.7–21.3)	21.2 (17.9–24.5)	16.6 (13.5–19.8)	6.3 (4.5–8.0)	10.0 (7.3–12.6)	—	4.1 (2.9–5.3)	5.1 (3.1–7.1)	3.1 (2.0–4.2)
Arizona (n = 10,952)	14.7 (13.5–15.9)	17.5 (15.5–19.4)	12.1 (10.6–13.6)	2.8 (2.2–3.3)	4.8 (3.8–5.8)	0.9 (0.4–1.3)	5.3 (4.5–6.1)	6.2 (4.9–7.5)	4.4 (3.5–5.4)
Arkansas (n = 5,298)	23.6 (21.3–25.8)	24.8 (21.4–28.2)	22.5 (19.5–25.5)	7.8 (6.2–9.3)	13.9 (11.0–16.8)	2.0 (0.9–3.0)	5.8 (4.5–7.1)	7.1 (4.9–9.3)	4.6 (3.2–6.0)
California (n = 11,393)	11.0 (10.2–11.7)	13.8 (12.6–15.0)	8.2 (7.3–9.1)	1.7 (1.4–2.0)	3.2 (2.5–3.8)	0.3 (0.1–0.5)	3.2 (2.8–3.7)	4.3 (3.6–5.1)	2.1 (1.6–2.6)
Colorado (n = 14,958)	15.6 (14.7–16.5)	17.7 (16.4–19.0)	13.5 (12.4–14.7)	3.7 (3.2–4.1)	7.0 (6.1–7.9)	0.4 (0.2–0.6)	5.2 (4.7–5.8)	6.1 (5.3–7.0)	4.3 (3.6–5.1)
Connecticut (n = 11,041)	13.3 (12.4–14.3)	14.8 (13.3–16.4)	12.0 (10.7–13.2)	2.0 (1.5–2.5)	3.4 (2.5–4.3)	0.7 (0.4–1.1)	4.1 (3.4–4.8)	5.3 (4.2–6.5)	3.0 (2.2–3.7)
Delaware (n = 4,057)	17.7 (16.0–19.4)	19.0 (16.3–21.7)	16.4 (14.3–18.6)	2.2 (1.6–2.9)	3.4 (2.2–4.5)	1.2 (0.6–1.8)	4.0 (3.1–4.9)	5.4 (3.9–6.9)	2.8 (1.7–3.9)
District of Columbia (n = 3,852)	14.7 (13.2–16.1)	17.7 (15.3–20.1)	12.1 (10.4–13.8)	1.3 (0.7–1.8)	1.8 (0.8–2.7)	—	2.4 (1.7–3.1)	3.6 (2.3–4.9)	1.4 (0.7–2.0)
Florida (n = 36,955)	15.5 (14.7–16.3)	17.8 (16.6–19.0)	13.3 (12.3–14.3)	3.0 (2.7–3.4)	4.8 (4.2–5.5)	1.3 (0.9–1.8)	4.7 (4.2–5.2)	5.6 (4.8–6.4)	3.9 (3.3–4.5)
Georgia (n = 5,381)	17.9 (16.5–19.3)	21.2 (18.9–23.5)	14.8 (13.1–16.5)	3.8 (3.0–4.5)	6.2 (4.8–7.7)	1.4 (0.9–2.0)	4.8 (3.9–5.7)	5.9 (4.5–7.4)	3.7 (2.7–4.7)
Hawaii (n = 8,087)	13.1 (12.0–14.2)	15.1 (13.4–16.8)	10.9 (9.6–12.3)	2.4 (1.9–2.9)	3.4 (2.6–4.2)	1.5 (0.9–2.1)	4.3 (3.6–5.0)	6.0 (4.8–7.1)	2.6 (1.9–3.3)
Idaho (n = 5,258)	14.5 (13.0–15.9)	14.7 (12.5–16.8)	14.3 (12.3–16.3)	6.1 (4.9–7.2)	11.1 (9.0–13.2)	1.1 (0.5–1.8)	4.6 (3.6–5.6)	4.7 (3.2–6.2)	4.5 (3.2–5.8)
Illinois (n = 4,764)	15.8 (14.4–17.2)	18.7 (16.5–20.8)	13.0 (11.3–14.8)	2.8 (2.1–3.4)	5.0 (3.8–6.2)	—	4.3 (3.5–5.1)	5.1 (3.8–6.4)	3.5 (2.5–4.5)
Indiana (n = 11,066)	21.1 (20.0–22.3)	23.6 (21.8–25.4)	18.8 (17.3–20.3)	4.1 (3.5–4.7)	7.6 (6.4–8.8)	0.7 (0.4–1.0)	4.7 (4.1–5.4)	5.0 (4.0–5.9)	4.5 (3.7–5.4)
Iowa (n = 7,257)	16.7 (15.5–18.0)	17.7 (15.9–19.5)	15.8 (14.2–17.4)	4.6 (3.9–5.3)	9.0 (7.6–10.3)	—	4.3 (3.6–5.0)	4.5 (3.5–5.5)	4.1 (3.2–5.1)
Kansas (n = 12,188)	17.2 (16.3–18.1)	18.7 (17.3–20.1)	15.7 (14.5–16.9)	5.9 (5.3–6.5)	11.2 (10.1–12.4)	0.8 (0.4–1.1)	4.9 (4.4–5.5)	6.0 (5.2–6.9)	3.8 (3.1–4.5)
Kentucky (n = 10,265)	24.5 (23.1–25.8)	25.0 (22.9–27.0)	24.0 (22.2–25.8)	7.4 (6.6–8.2)	13.6 (12.1–15.1)	1.6 (1.1–2.2)	5.6 (4.9–6.4)	6.3 (5.1–7.5)	5.0 (4.1–5.9)
Louisiana (n = 5,256)	22.8 (20.8–24.8)	25.5 (22.4–28.7)	20.2 (17.6–22.7)	5.1 (4.1–6.1)	9.1 (7.1–11.0)	1.3 (0.7–1.9)	6.0 (4.8–7.2)	7.4 (5.4–9.3)	4.7 (3.3–6.1)
Maine (n = 10,019)	19.8 (18.4–21.1)	21.6 (19.6–23.6)	18.0 (16.3–19.8)	2.8 (2.2–3.4)	4.8 (3.7–5.9)	1.0 (0.5–1.5)	3.8 (3.2–4.5)	5.0 (3.9–6.1)	2.7 (2.0–3.4)
Maryland (n = 18,473)	13.7 (12.9–14.5)	15.6 (14.3–17.0)	11.9 (10.9–12.9)	1.6 (1.3–1.9)	2.9 (2.3–3.5)	0.4 (0.2–0.6)	3.2 (2.8–3.7)	4.5 (3.7–5.3)	2.0 (1.6–2.5)
Massachusetts (n = 8,415)	13.6 (12.6–14.7)	15.5 (13.9–17.1)	11.9 (10.5–13.3)	2.0 (1.5–2.4)	3.1 (2.3–3.9)	1.0 (0.5–1.4)	4.3 (3.6–5.1)	5.8 (4.6–7.0)	2.9 (2.1–3.7)
Michigan (n = 12,024)	20.4 (19.4–21.4)	22.3 (20.8–23.8)	18.7 (17.4–20.0)	3.6 (3.1–4.0)	6.2 (5.4–7.1)	1.1 (0.7–1.5)	4.9 (4.4–5.5)	5.6 (4.7–6.5)	4.3 (3.6–5.0)
Minnesota (n = 16,831)	15.2 (14.5–15.9)	16.6 (15.5–17.6)	13.9 (12.9–14.8)	4.3 (3.9–4.7)	7.8 (7.0–8.5)	0.9 (0.7–1.2)	3.8 (3.4–4.1)	4.8 (4.2–5.4)	2.8 (2.3–3.2)
Mississippi (n = 5,135)	22.7 (21.0–24.4)	24.3 (21.7–27.0)	21.3 (19.1–23.5)	7.2 (6.2–8.3)	13.2 (11.2–15.2)	1.7 (1.0–2.4)	4.7 (3.8–5.6)	5.4 (3.9–6.9)	4.0 (2.9–5.1)
Missouri (n = 7,126)	22.1 (20.5–23.7)	24.5 (22.0–26.9)	19.9 (17.8–22.0)	4.6 (3.8–5.5)	8.8 (7.2–10.4)	—	5.0 (4.1–5.9)	5.7 (4.4–7.1)	4.3 (3.2–5.4)
Montana (n = 5,971)	18.5 (17.0–20.0)	19.8 (17.6–22.0)	17.3 (15.2–19.4)	7.7 (6.7–8.8)	14.0 (12.1–16.0)	1.5 (0.8–2.1)	4.1 (3.2–4.9)	4.3 (3.1–5.5)	3.8 (2.6–5.1)
Nebraska (n = 15,183)	17.0 (15.9–18.0)	18.6 (17.0–20.2)	15.4 (14.1–16.7)	5.7 (5.1–6.2)	10.5 (9.5–11.6)	0.9 (0.6–1.2)	4.9 (4.3–5.6)	5.3 (4.4–6.3)	4.5 (3.6–5.5)
Nevada (n = 4,344)	16.5 (14.8–18.1)	18.9 (16.3–21.5)	14.1 (12.0–16.1)	2.5 (1.9–3.1)	4.3 (3.2–5.4)	0.7 (0.3–1.1)	6.0 (4.9–7.2)	6.9 (5.2–8.7)	5.1 (3.7–6.5)
New Hampshire (n = 6,420)	18.0 (16.4–19.6)	18.5 (16.3–20.7)	17.6 (15.2–19.9)	2.2 (1.6–2.8)	3.8 (2.6–4.9)	—	5.1 (4.1–6.1)	6.8 (5.2–8.3)	3.5 (2.1–4.8)
New Jersey (n = 7,652)	14.0 (12.6–15.3)	14.9 (12.9–17.0)	13.1 (11.4–14.8)	2.3 (1.6–2.9)	3.7 (2.4–4.9)	1.0 (0.5–1.4)	3.7 (2.9–4.5)	4.5 (3.2–5.7)	3.0 (2.1–3.9)
New Mexico (n = 6,024)	16.6 (15.1–18.1)	19.4 (16.9–21.8)	14.0 (12.2–15.8)	3.9 (3.2–4.6)	7.1 (5.8–8.5)	0.9 (0.5–1.3)	4.9 (3.8–5.9)	5.8 (4.1–7.5)	3.9 (2.7–5.1)
New York (n = 34,190)	14.2 (13.4–14.9)	16.7 (15.4–17.9)	11.9 (11.0–12.8)	2.2 (1.9–2.5)	3.5 (2.9–4.0)	1.1 (0.8–1.5)	4.1 (3.6–4.6)	5.4 (4.6–6.2)	2.9 (2.4–3.5)
North Carolina (n = 6,536)	17.9 (16.7–19.1)	20.7 (18.8–22.5)	15.3 (13.7–16.9)	4.0 (3.4–4.7)	7.3 (6.1–8.5)	1.0 (0.6–1.4)	4.4 (3.7–5.1)	5.4 (4.3–6.5)	3.4 (2.6–4.2)
North Dakota (n = 5,742)	19.8 (18.3–21.2)	22.3 (20.3–24.4)	17.1 (15.0–19.2)	7.1 (6.1–8.1)	12.6 (10.9–14.4)	1.4 (0.8–2.0)	3.6 (2.8–4.5)	3.9 (2.8–5.0)	3.4 (2.2–4.6)
Ohio (n = 12,389)	22.5 (21.3–23.8)	24.7 (22.7–26.6)	20.5 (18.9–22.1)	4.7 (4.1–5.3)	9.0 (7.7–10.2)	0.8 (0.4–1.1)	5.7 (5.0–6.4)	5.8 (4.7–6.9)	5.6 (4.6–6.6)
Oklahoma (n = 6,925)	19.6 (18.2–21.0)	21.4 (19.1–23.7)	17.9 (16.1–19.6)	6.0 (5.2–6.9)	11.4 (9.7–13.1)	0.9 (0.5–1.3)	6.7 (5.7–7.7)	7.9 (6.1–9.6)	5.5 (4.5–6.5)
Oregon (n = 5,439)	16.2 (14.9–17.5)	18.1 (16.1–20.1)	14.4 (12.8–16.0)	4.0 (3.3–4.7)	7.2 (5.9–8.5)	0.9 (0.5–1.4)	4.4 (3.7–5.2)	5.8 (4.5–7.1)	3.1 (2.3–3.9)
Pennsylvania (n = 6,810)	18.0 (16.7–19.2)	20.1 (18.1–22.0)	16.0 (14.4–17.6)	4.1 (3.4–4.7)	7.9 (6.7–9.1)	—	4.2 (3.5–4.8)	4.7 (3.7–5.7)	3.6 (2.8–4.4)
Rhode Island (n = 5,457)	14.4 (13.0–15.8)	16.9 (14.6–19.2)	12.2 (10.6–13.8)	1.5 (1.0–2.0)	2.8 (1.8–3.7)	—	4.5 (3.5–5.4)	5.9 (4.2–7.7)	3.2 (2.2–4.1)
South Carolina (n = 11,236)	20.0 (18.9–21.2)	22.9 (21.0–24.7)	17.4 (16.0–18.8)	3.7 (3.1–4.3)	6.8 (5.7–8.0)	0.9 (0.6–1.2)	4.8 (4.1–5.4)	5.2 (4.2–6.2)	4.3 (3.5–5.1)
South Dakota (n = 5,767)	18.1 (16.3–20.0)	21.1 (18.2–24.1)	15.1 (12.9–17.3)	5.9 (4.7–7.0)	10.9 (8.7–13.1)	—	2.9 (2.1–3.7)	3.3 (2.1–4.5)	2.5 (1.5–3.5)
Tennessee (n = 6,167)	22.1 (20.5–23.7)	23.9 (21.4–26.4)	20.4 (18.4–22.3)	6.8 (5.8–7.7)	12.4 (10.6–14.3)	1.5 (0.9–2.0)	5.7 (4.8–6.6)	7.3 (5.7–8.9)	4.2 (3.2–5.2)
Texas (n = 11,709)	14.3 (13.1–15.5)	17.3 (15.4–19.2)	11.3 (9.8–12.7)	4.3 (3.6–5.1)	7.4 (6.0–8.7)	1.3 (0.7–2.0)	4.7 (3.9–5.5)	6.7 (5.3–8.2)	2.7 (2.0–3.5)
Utah (n = 10,988)	8.8 (8.0–9.6)	10.4 (9.1–11.7)	7.1 (6.1–8.1)	3.4 (2.9–4.0)	5.9 (5.0–6.9)	0.9 (0.5–1.3)	5.1 (4.4–5.7)	5.8 (4.9–6.8)	4.3 (3.4–5.2)
Vermont (n = 6,540)	17.0 (15.6–18.4)	19.5 (17.3–21.7)	14.7 (12.9–16.5)	3.1 (2.5–3.8)	5.9 (4.5–7.2)	—	3.4 (2.6–4.2)	4.5 (3.2–5.9)	2.3 (1.5–3.1)
Virginia (n = 9,002)	15.3 (14.3–16.3)	17.0 (15.4–18.5)	13.7 (12.5–15.0)	3.7 (3.2–4.2)	6.6 (5.7–7.6)	0.9 (0.6–1.3)	4.9 (4.3–5.6)	6.1 (5.1–7.2)	3.7 (2.9–4.5)
Washington (n = 14,259)	13.9 (13.1–14.7)	16.0 (14.7–17.2)	11.9 (10.9–13.0)	3.5 (3.0–3.9)	6.3 (5.5–7.1)	0.7 (0.4–1.0)	5.3 (4.7–5.8)	6.7 (5.8–7.5)	3.9 (3.2–4.5)
West Virginia (n = 7,151)	24.8 (23.6–26.1)	25.8 (23.9–27.7)	23.9 (22.2–25.5)	8.5 (7.7–9.4)	15.9 (14.3–17.5)	1.5 (1.0–2.0)	4.7 (4.0–5.3)	4.9 (3.9–5.9)	4.4 (3.5–5.3)
Wisconsin (n = 5,271)	17.1 (15.6–18.5)	18.0 (15.8–20.2)	16.2 (14.1–18.2)	4.4 (3.6–5.2)	8.0 (6.5–9.6)	0.9 (0.4–1.4)	5.3 (4.3–6.3)	6.7 (5.0–8.3)	4.0 (2.9–5.1)
Wyoming (n = 4,497)	18.9 (17.0–20.9)	18.8 (16.0–21.6)	19.1 (16.4–21.9)	9.8 (8.2–11.3)	17.0 (14.3–19.8)	2.3 (1.0–3.6)	5.5 (4.3–6.8)	6.1 (4.3–7.8)	5.0 (3.3–6.7)

Abbreviations: CI, confidence interval; —, estimates not presented because of relative standard error >30%.

a Persons aged ≥18 years who reported having smoked ≥100 cigarettes during their lifetime and smoked every day or some days at the time of survey. Excludes respondents whose smoking status was unknown.

b Persons aged ≥18 years who reported currently using chewing tobacco, snuff, or snus every day or some days at the time of survey. Excludes respondents whose using status was unknown.

c Persons aged ≥18 years who reported currently using e-cigarette every day or some days at the time of survey. Excludes respondents whose using status was unknown.

### Smokeless tobacco

Prevalence of current smokeless tobacco use ranged from 1.3% (DC) to 9.8% (Wyoming) (Table 1). Prevalence of current smokeless tobacco use was significantly higher among men than women in 41 states for which statistically stable estimates among women could be computed. Prevalence ranged from 1.8% (DC) to 17.0% (Wyoming) among men and from 0.3% (California) to 2.3% (Wyoming) among women. Five of the 10 states with the highest prevalence of current smokeless tobacco use were in the South (West Virginia [8.5], Arkansas [7.8], Kentucky [7.4], Mississippi [7.2], and Tennessee [6.8]), 4 states were in the West (Wyoming [9.8], Montana [7.7], Alaska [6.3], and Idaho [6.1]), and 1 state was in the Midwest (North Dakota [7.1]).

### E-cigarettes

Prevalence of ever e-cigarette use ranged from 16.2% (DC) to 28.4% (Arkansas) (Table 2), and was significantly higher among men than women in 47 states and DC. Prevalence also differed significantly by race/ethnicity in 41 states and DC for which statistically stable estimates could be computed. The prevalence of ever use of e-cigarettes ranged from 20.5% (DC) to 30.4% (Alaska) among men and from 12.5% (DC) to 26.9% (Arkansas) among women. Prevalence of ever e-cigarette use ranged from 16.4% (DC) to 30.1% (Arkansas) among non-Hispanic whites, from 14.4% (Mississippi) to 38.5% (New Mexico) among non-Hispanic blacks, from 12.4% (North Carolina) to 43.4% (Alaska) among Hispanics, and from 14.2% (Maryland) to 42.7% (Mississippi) among non-Hispanics of other races.

**Table 2:** State-Specific Prevalence of Ever E-cigarette Use^a^ Among Adults Aged 18 Years or Older, by Sex and Race/Ethnicity — Behavioral Risk Factor Surveillance System, United States, 2016

State	Total % (95% CI)	Sex		Race/Ethnicity			
		Men % (95% CI)	Women % (95% CI)	Non-Hispanic White % (95% CI)	Non-Hispanic Black % (95% CI)	Hispanic % (95% CI)	Non-Hispanic Other % (95% CI)
All 50 states and District of Columbia median	22.1 (20.4–23.7)	24.8 (21.9–27.8)	18.6 (16.6–20.7)	22.4 (20.7–24.1)	21.9 (17.3–26.5)	20.8 (18.0–23.6)	26.3 (18.8–33.8)
Alabama	24.7 (23.2–26.2)	27.7 (25.3–30.1)	21.9 (20.1–23.7)	26.3 (24.4–28.1)	17.4 (15.0–19.8)	30.5 (18.5–42.6)	39.8 (30.8–48.8)
Alaska	24.7 (22.0–27.4)	30.4 (26.2–34.5)	18.4 (15.3–21.5)	21.8 (19.0–24.6)	—	43.4 (27.0–59.8)	30.9 (24.6–37.2)
Arizona	23.2 (21.7–24.7)	27.4 (25.0–29.8)	19.2 (17.3–21.1)	23.6 (21.8–25.3)	29.6 (20.6–38.6)	21.8 (18.1–25.4)	22.5 (17.8–27.2)
Arkansas	28.4 (25.9–30.9)	30.0 (26.3–33.7)	26.9 (23.5–30.2)	30.1 (27.4–32.9)	17.1 (10.9–23.3)	26.6 (13.8–39.4)	34.4 (21.6–47.3)
California	20.5 (19.5–21.5)	25.2 (23.6–26.7)	16.0 (14.7–17.3)	23.1 (21.6–24.6)	24.3 (19.3–29.4)	18.7 (17.0–20.4)	16.3 (13.8–18.8)
Colorado	23.1 (22.0–24.1)	25.7 (24.1–27.2)	20.6 (19.1–22.0)	22.7 (21.5–23.9)	26.4 (19.8–33.0)	23.1 (20.5–25.7)	27.7 (22.4–33.1)
Connecticut	17.8 (16.6–19.0)	21.4 (19.5–23.3)	14.4 (13.0–15.8)	17.9 (16.5–19.3)	14.9 (11.1–18.7)	20.0 (16.5–23.5)	18.8 (13.8–23.8)
Delaware	19.0 (17.1–20.9)	21.3 (18.6–24.1)	16.9 (14.3–19.6)	20.0 (17.6–22.4)	15.8 (11.7–19.9)	12.5 (7.2–17.7)	24.2 (16.2–32.1)
District of Columbia	16.2 (14.5–17.9)	20.5 (17.7–23.3)	12.5 (10.5–14.5)	16.4 (13.4–19.4)	15.5 (13.5–17.5)	15.8 (9.4–22.2)	22.4 (14.8–30.1)
Florida	20.3 (19.4–21.2)	23.2 (21.8–24.6)	17.6 (16.4–18.8)	22.7 (21.6–23.8)	15.8 (13.1–18.5)	17.5 (15.3–19.7)	16.0 (12.7–19.3)
Georgia	20.8 (19.3–22.4)	23.2 (20.8–25.7)	18.6 (16.6–20.7)	23.6 (21.5–25.7)	18.2 (15.3–21.1)	13.9 (9.0–18.8)	20.2 (13.1–27.4)
Hawaii	22.2 (20.8–23.6)	26.1 (23.9–28.2)	18.3 (16.5–20.0)	21.4 (18.6–24.3)	26.6 (12.8–40.4)	37.1 (31.3–43.0)	20.3 (18.7–21.9)
Idaho	22.5 (20.7–24.4)	24.3 (21.4–27.3)	20.8 (18.4–23.1)	22.2 (20.2–24.1)	—	20.4 (13.6–27.3)	38.1 (26.9–49.3)
Illinois	21.2 (19.6–22.8)	26.2 (23.7–28.7)	16.5 (14.5–18.4)	21.9 (19.9–23.8)	22.8 (18.2–27.4)	16.7 (12.7–20.7)	20.9 (14.6–27.3)
Indiana	24.5 (23.1–25.8)	26.9 (24.9–29.0)	22.1 (20.4–23.8)	24.6 (23.2–26.0)	22.9 (17.9–27.9)	19.0 (13.9–24.2)	30.5 (22.4–38.5)
Iowa	19.2 (17.8–20.5)	22.6 (20.5–24.7)	15.8 (14.1–17.5)	19.0 (17.6–20.4)	21.8 (10.3–33.2)	17.8 (10.6–24.9)	24.2 (15.5–32.9)
Kansas	24.2 (23.1–25.3)	27.5 (25.8–29.1)	20.9 (19.5–22.3)	23.3 (22.2–24.4)	27.2 (21.1–33.3)	22.9 (18.8–27.1)	36.1 (30.3–42.0)
Kentucky	26.9 (25.5–28.4)	27.9 (25.7–30.0)	26.1 (24.2–27.9)	27.0 (25.5–28.4)	21.9 (17.3–26.5)	36.8 (23.2–50.3)	33.6 (25.3–41.8)
Louisiana	24.5 (22.4–26.6)	27.7 (24.4–30.9)	21.5 (18.8–24.2)	27.9 (25.3–30.6)	16.2 (12.6–19.8)	27.6 (15.0–40.2)	32.7 (21.5–43.8)
Maine	21.4 (20.0–22.8)	24.5 (22.3–26.6)	18.5 (16.7–20.4)	21.0 (19.5–22.4)	—	—	40.7 (31.8–49.6)
Maryland	18.1 (17.1–19.1)	20.9 (19.3–22.4)	15.6 (14.3–16.8)	20.4 (19.1–21.7)	16.0 (14.0–17.9)	15.5 (11.8–19.2)	14.2 (10.9–17.6)
Massachusetts	18.5 (17.3–19.7)	21.4 (19.5–23.3)	15.7 (14.1–17.4)	18.9 (17.4–20.3)	16.7 (11.7–21.7)	15.3 (11.9–18.6)	18.8 (13.9–23.7)
Michigan	23.0 (22.0–24.1)	25.8 (24.2–27.4)	20.5 (19.1–21.8)	23.1 (21.9–24.3)	20.9 (17.8–23.9)	27.4 (21.2–33.5)	24.9 (20.2–29.6)
Minnesota	19.6 (18.8–20.4)	22.5 (21.3–23.7)	16.8 (15.7–17.8)	19.2 (18.3–20.0)	23.4 (18.7–28.0)	15.4 (12.0–18.8)	24.4 (20.6–28.1)
Mississippi	22.2 (20.5–24.0)	25.1 (22.3–27.8)	19.6 (17.4–21.8)	25.8 (23.4–28.1)	14.4 (12.1–16.8)	29.1 (14.4–43.8)	42.7 (27.9–57.5)
Missouri	25.2 (23.5–27.0)	27.9 (25.3–30.4)	22.7 (20.3–25.1)	25.3 (23.4–27.1)	21.9 (15.6–28.3)	26.6 (14.3–38.9)	34.3 (25.0–43.5)
Montana	21.8 (20.2–23.5)	24.4 (21.9–26.8)	19.3 (17.0–21.6)	20.7 (18.9–22.4)	—	28.2 (16.7–39.8)	34.2 (27.6–40.7)
Nebraska	22.6 (21.4–23.8)	25.1 (23.3–26.9)	20.2 (18.6–21.8)	22.2 (20.9–23.4)	27.0 (18.1–35.9)	17.9 (14.2–21.7)	35.8 (28.4–43.2)
Nevada	23.2 (21.2–25.1)	26.4 (23.5–29.2)	20.0 (17.4–22.5)	24.3 (21.8–26.8)	26.0 (18.2–33.7)	17.6 (14.3–21.0)	27.8 (20.5–35.1)
New Hampshire	21.2 (19.4–22.9)	24.7 (22.1–27.3)	17.8 (15.5–20.1)	20.7 (19.0–22.5)	—	22.9 (9.5–36.4)	30.4 (20.4–40.3)
New Jersey	18.2 (16.6–19.8)	21.6 (19.0–24.2)	15.0 (13.2–16.9)	20.2 (18.0–22.3)	20.7 (15.7–25.7)	13.3 (10.2–16.3)	14.5 (9.6–19.3)
New Mexico	25.5 (23.7–27.4)	29.1 (26.2–32.0)	22.1 (19.7–24.5)	23.4 (21.0–25.9)	38.5 (19.0–58.0)	27.6 (24.5–30.8)	23.4 (18.1–28.8)
New York	19.7 (18.8–20.7)	24.0 (22.5–25.4)	15.9 (14.7–17.0)	21.3 (20.2–22.5)	17.6 (14.9–20.3)	18.8 (16.5–21.2)	15.4 (12.1–18.7)
North Carolina	21.2 (19.9–22.5)	24.2 (22.2–26.2)	18.4 (16.7–20.2)	22.4 (20.7–24.1)	19.7 (16.8–22.6)	12.4 (8.7–16.2)	23.0 (16.8–29.2)
North Dakota	22.1 (20.4–23.7)	24.6 (22.3–26.9)	19.4 (17.0–21.7)	20.4 (18.8–22.1)	26.2 (11.3–41.1)	36.9 (20.7–53.2)	36.0 (28.3–43.8)
Ohio	25.0 (23.7–26.4)	27.0 (25.0–29.0)	23.2 (21.5–25.0)	24.7 (23.3–26.2)	25.5 (21.0–30.0)	25.9 (17.2–34.6)	31.3 (24.2–38.4)
Oklahoma	27.9 (26.3–29.5)	30.1 (27.6–32.7)	25.7 (23.7–27.7)	27.0 (25.2–28.8)	26.0 (19.3–32.8)	25.6 (19.2–31.9)	33.8 (29.1–38.5)
Oregon	20.8 (19.4–22.2)	23.2 (21.1–25.3)	18.5 (16.6–20.3)	20.7 (19.2–22.2)	30.2 (13.3–47.0)	17.2 (12.3–22.2)	24.3 (18.5–30.2)
Pennsylvania	21.2 (19.8–22.5)	24.0 (21.9–26.0)	18.5 (16.7–20.3)	20.6 (19.1–22.1)	21.2 (16.6–25.8)	28.8 (21.2–36.4)	19.6 (14.2–24.9)
Rhode Island	20.9 (19.1–22.7)	24.8 (21.9–27.8)	17.3 (15.1–19.4)	20.8 (18.7–22.8)	22.2 (13.5–30.9)	20.6 (15.2–26.0)	22.3 (14.0–30.5)
South Carolina	22.9 (21.6–24.1)	26.6 (24.6–28.6)	19.4 (17.9–20.9)	23.9 (22.4–25.4)	18.2 (15.7–20.6)	29.1 (20.8–37.4)	30.2 (23.6–36.7)
South Dakota	20.3 (18.4–22.2)	23.9 (20.9–26.9)	16.7 (14.3–19.2)	18.8 (16.8–20.7)	—	—	35.6 (27.4–43.9)
Tennessee	25.1 (23.3–26.8)	29.0 (26.3–31.7)	21.4 (19.2–23.5)	25.4 (23.6–27.3)	21.1 (16.3–25.9)	26.1 (15.4–36.8)	28.0 (19.1–36.9)
Texas	23.7 (22.1–25.2)	29.2 (26.8–31.6)	18.2 (16.3–20.2)	25.8 (23.7–27.9)	22.4 (17.2–27.6)	20.8 (18.0–23.6)	25.8 (18.7–32.9)
Utah	19.1 (18.0–20.2)	22.5 (20.8–24.2)	15.7 (14.2–17.2)	17.8 (16.6–19.0)	—	24.3 (20.4–28.3)	27.2 (20.6–33.9)
Vermont	18.7 (17.2–20.3)	22.7 (20.3–25.1)	15.0 (13.1–16.9)	18.2 (16.6–19.8)	—	28.9 (14.3–43.5)	28.3 (18.8–37.9)
Virginia	21.5 (20.4–22.7)	24.5 (22.7–26.3)	18.7 (17.1–20.3)	22.6 (21.2–24.1)	19.4 (16.6–22.1)	17.7 (13.6–21.7)	21.8 (16.7–26.9)
Washington	22.5 (21.5–23.5)	26.5 (25.0–28.0)	18.6 (17.3–19.9)	22.7 (21.6–23.8)	22.0 (15.9–28.0)	19.9 (16.5–23.2)	24.3 (21.0–27.7)
West Virginia	23.1 (21.9–24.4)	24.2 (22.3–26.1)	22.1 (20.4–23.7)	23.1 (21.8–24.4)	27.4 (19.3–35.5)	—	26.3 (18.8–33.8)
Wisconsin	21.8 (20.0–23.5)	24.4 (21.9–27.0)	19.1 (16.8–21.4)	21.7 (19.9–23.5)	19.2 (11.6–26.8)	20.3 (10.4–30.3)	25.0 (17.0–33.0)
Wyoming	24.3 (22.1–26.4)	27.7 (24.4–30.9)	20.7 (17.9–23.5)	23.1 (20.8–25.3)	—	27.6 (19.1–36.1)	37.3 (23.4–51.2)

Abbreviations: CI, confidence interval; — , estimates not presented because of relative standard error >30%.

a Persons aged ≥18 years who reported having ever used an e-cigarette or other electronic “vaping” product, even just 1 time, during their lifetime. Excludes respondents whose ever using status was unknown.

Prevalence of current e-cigarette use ranged from 2.4% (DC) to 6.7% (Oklahoma) (Table 1). The prevalence of current e-cigarette use was significantly higher among men than among women in 30 of 50 states and DC. Prevalence ranged from 3.3% (South Dakota) to 7.9% (Oklahoma) among men and 1.4% (DC) to 5.6% (Ohio) among women. Five of the 10 states with the highest prevalence of current e-cigarette smoking were in the South (Oklahoma [6.7], Louisiana [6.0], Arkansas [5.8], Tennessee [5.7], and Kentucky [5.6]); 3 states were in the West (Nevada [6.0], Wyoming [5.5], and Arizona [5.3]); and 2 states were in the Midwest (Ohio [5.7] and Wisconsin [5.3]).

### Cigarette smoking among current e-cigarette users

Among current adult e-cigarette users, the prevalence of current cigarette smoking was significantly higher than the prevalence of former cigarette smoking or never cigarette smoking in all 50 states and DC (Table 3). Among current e-cigarette users, the prevalence of current cigarette smoking ranged from 36.7% (Utah) to 72.1% (Missouri); the prevalence of former cigarette smokers ranged from 19.3% (Missouri) to 44.8% (Alaska); and the prevalence of never cigarette smokers ranged from 7.1% (Maine) to 32.2% (California). More than half of current e-cigarette users were current cigarette smokers in 39 states, ranging from 50.6% (Rhode Island) to 72.1% (Missouri).

**Table 3:** State-Specific Percentage of Cigarette Smoking Status Among Current E-cigarette Users^a^ Aged 18 Years or Older — Behavioral Risk Factor Surveillance System, United States, 2016

State	Percentage of Current Cigarette Smokers^b^, % (95% CI)	Percentage of Former Cigarette Smokers^c^, % (95% CI)	Percentage of Never-Cigarette Smokers^d^, % (95% CI)
Alabama	60.5 (53.1–67.9)	27.0 (20.3–33.8)	12.5 (7.1–17.9)
Alaska	49.2 (34.7–63.8)	44.8 (29.7–59.9)	—
Arizona	46.3 (38.6–53.9)	34.6 (27.1–42.1)	19.1 (12.3–26.0)
Arkansas	55.4 (43.9–67.0)	26.4 (16.9–35.9)	18.1 (8.0–28.2)
California	38.3 (31.7–44.9)	29.4 (23.0–35.8)	32.2 (25.3–39.2)
Colorado	53.9 (48.2–59.7)	25.4 (20.6–30.2)	20.6 (15.5–25.7)
Connecticut	52.2 (43.7–60.7)	26.0 (19.1–32.9)	21.8 (13.9–29.7)
Delaware	54.1 (42.6–65.6)	24.8 (15.2–34.3)	21.1 (10.7–31.6)
District of Columbia	46.5 (31.6–61.3)	23.2 (10.4–36.0)	30.3 (15.6–45.0)
Florida	49.0 (43.7–54.3)	31.2 (26.4–36.0)	19.8 (14.9–24.8)
Georgia	53.2 (43.9–62.6)	29.7 (21.0–38.3)	17.1 (9.6–24.6)
Hawaii	44.1 (36.1–52.0)	31.0 (23.7–38.3)	25.0 (17.4–32.5)
Idaho	51.0 (40.0–62.0)	34.4 (24.1–44.7)	14.6 (6.6–22.5)
Illinois	55.5 (45.7–65.3)	29.3 (20.0–38.6)	15.2 (8.5–21.9)
Indiana	58.5 (51.5–65.6)	28.7 (22.4–35.0)	12.7 (7.5–18.0)
Iowa	60.2 (52.0–68.5)	27.2 (19.7–34.6)	12.6 (6.8–18.4)
Kansas	56.1 (50.0–62.2)	27.4 (22.2–32.5)	16.5 (10.9–22.1)
Kentucky	51.6 (44.7–58.5)	32.9 (26.4–39.4)	15.5 (9.0–22.0)
Louisiana	58.9 (48.7–69.1)	25.7 (16.8–34.6)	15.4 (7.8–23.0)
Maine	64.6 (56.3–73.0)	28.2 (20.2–36.3)	7.1 (3.2–11.0)
Maryland	49.8 (42.3–57.3)	33.2 (26.1–40.3)	17.0 (11.0–22.9)
Massachusetts	51.3 (42.7–59.9)	27.5 (19.5–35.6)	21.2 (13.5–28.8)
Michigan	57.2 (51.2–63.2)	21.6 (17.2–26.0)	21.2 (15.3–27.0)
Minnesota	47.5 (42.2–52.9)	28.6 (24.0–33.1)	23.9 (18.8–29.0)
Mississippi	56.3 (46.1–66.5)	21.4 (13.4–29.5)	22.3 (12.9–31.6)
Missouri	72.1 (64.3–79.9)	19.3 (12.5–26.2)	8.6 (4.0–13.1)
Montana	62.1 (51.3–72.9)	25.7 (16.1–35.2)	—
Nebraska	59.5 (52.4–66.6)	21.5 (16.0–26.9)	19.0 (12.4–25.7)
Nevada	51.3 (41.6–61.0)	28.6 (20.1–37.0)	20.1 (11.9–28.3)
New Hampshire	66.7 (57.4–75.9)	21.3 (13.4–29.1)	12.1 (5.8–18.3)
New Jersey	54.9 (44.1–65.6)	24.8 (16.0–33.7)	20.3 (11.0–29.6)
New Mexico	50.9 (39.9–62.0)	25.6 (15.8–35.3)	23.5 (14.7–32.3)
New York	48.5 (42.6–54.4)	27.4 (22.2–32.7)	24.1 (18.6–29.6)
North Carolina	52.4 (44.5–60.4)	33.2 (25.5–40.8)	14.4 (8.9–19.8)
North Dakota	64.9 (54.2–75.6)	25.7 (16.3–35.1)	—
Ohio	65.6 (59.4–71.8)	21.1 (16.0–26.2)	13.3 (8.8–17.9)
Oklahoma	54.5 (46.8–62.2)	31.7 (24.9–38.4)	13.9 (7.5–20.2)
Oregon	65.0 (56.9–73.0)	24.6 (17.6–31.6)	10.5 (5.2–15.7)
Pennsylvania	58.4 (50.7–66.0)	27.0 (19.9–34.1)	14.6 (9.1–20.1)
Rhode Island	50.6 (39.4–61.9)	31.0 (21.2–40.7)	18.4 (8.4–28.3)
South Carolina	58.9 (52.0–65.7)	27.9 (21.8–34.1)	13.2 (8.2–18.3)
South Dakota	70.5 (58.2–82.9)	20.0 (8.5–31.5)	—
Tennessee	63.0 (54.8–71.3)	22.9 (16.0–29.8)	14.0 (7.4–20.7)
Texas	51.3 (42.5–60.0)	37.3 (28.5–46.0)	11.4 (6.2–16.7)
Utah	36.7 (30.5–42.9)	35.1 (28.9–41.3)	28.2 (22.0–34.4)
Vermont	53.0 (41.1–64.8)	19.8 (12.4–27.2)	27.2 (14.9–39.6)
Virginia	45.0 (38.3–51.7)	30.6 (24.2–36.9)	24.4 (18.2–30.7)
Washington	45.2 (39.8–50.6)	33.1 (28.1–38.0)	21.7 (16.8–26.5)
West Virginia	63.5 (56.4–70.7)	29.9 (23.2–36.7)	—
Wisconsin	56.6 (46.7–66.5)	23.9 (16.2–31.7)	19.5 (10.3–28.6)
Wyoming	59.4 (48.1–70.7)	23.1 (13.9–32.3)	17.5 (8.2–26.8)

Abbreviations: CI, confidence interval; —, estimates not presented because of relative standard error >30%.

a Persons aged ≥18 years who reported currently using e-cigarette every day or some days. Respondents with unknown current cigarette smoking status (0.5% of current e-cigarette users) were excluded.

b Persons aged ≥18 years who reported currently using e-cigarette every day or some days and also reported having smoked ≥100 cigarettes during their lifetime and smoked every day or some days at the time of survey.

c Persons aged ≥18 years who reported currently using e-cigarette every day or some days and reported having smoked ≥100 cigarettes during their lifetime but did not smoke every day or some days at the time of survey.

d Persons aged ≥18 years who reported currently using e-cigarette every day or some days and reported never having smoked or having smoked <100 cigarettes during their lifetime at the time of survey.

Of the 12 states (Alabama, Arkansas, Indiana, Kentucky, Louisiana, Michigan, Mississippi, Missouri, Ohio, South Carolina, Tennessee, and West Virginia) that were in the highest quartile of current cigarette smoking prevalence (prevalences of 19.9%–24.8%), 5 of these states (Arkansas, Kentucky, Louisiana, Ohio, and Tennessee) also were in the highest quartile of current e-cigarette use (prevalences of 5.3%–6.7%). Four of the 12 states (Alabama, Michigan, Missouri, and South Carolina) were in the second-highest quartile of current e-cigarette use (prevalences of 4.8%–5.2%); and 3 of the 12 states (Indiana, Mississippi, and West Virginia) were in the third-highest quartile of current e-cigarette use (prevalences of 4.2%–4.7%) (Figure).

**Figure:**
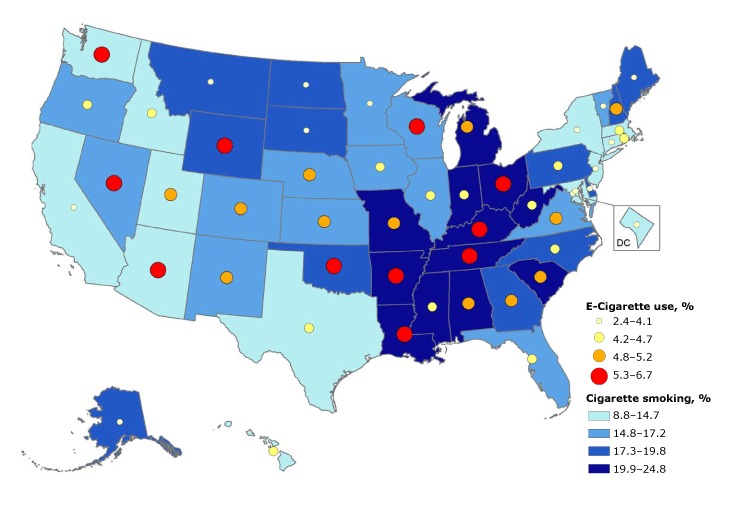
State-specific prevalence of current cigarette smoking and e-cigarette use among adults aged 18 years or older, by percentile — Behavioral Risk Factor Surveillance System, United States, 2016. Current cigarette smoking was defined as persons aged 18 years or older who reported having smoked 100 or more cigarettes during their lifetime and smoked every day or some days at the time of survey; it excludes respondents whose smoking status was unknown. E-cigarette use was defined as persons aged 18 years or older who reported currently using e-cigarettes every day or some days at the time of survey; it excludes respondents whose using status was unknown.

**Table:** 

State	Cigarette smoking, %	E-cigarette Use, %
Alabama	21.5	5.1
Alaska	19.0	4.1
Arizona	14.7	5.3
Arkansas	23.6	5.8
California	11.0	3.2
Colorado	15.6	5.2
Connecticut	13.3	4.1
Delaware	17.7	4.0
District of Columbia	14.7	2.4
Florida	15.5	4.7
Georgia	17.9	4.8
Hawaii	13.1	4.3
Idaho	14.5	4.6
Illinois	15.8	4.3
Indiana	21.1	4.7
Iowa	16.7	4.3
Kansas	17.2	4.9
Kentucky	24.5	5.6
Louisiana	22.8	6.0
Maine	19.8	3.8
Maryland	13.7	3.2
Massachusetts	13.6	4.3
Michigan	20.4	4.9
Minnesota	15.2	3.8
Mississippi	22.7	4.7
Missouri	22.1	5.0
Montana	18.5	4.1
Nebraska	17.0	4.9
Nevada	16.5	6.0
New Hampshire	18.0	5.1
New Jersey	14.0	3.7
New Mexico	16.6	4.9
New York	14.2	4.1
North Carolina	17.9	4.4
North Dakota	19.8	3.6
Ohio	22.5	5.7
Oklahoma	19.6	6.7
Oregon	16.2	4.4
Pennsylvania	18.0	4.2
Rhode Island	14.4	4.5
South Carolina	20.0	4.8
South Dakota	18.1	2.9
Tennessee	22.1	5.7
Texas	14.3	4.7
Utah	8.8	5.1
Vermont	17.0	3.4
Virginia	15.3	4.9
Washington	13.9	5.3
West Virginia	24.8	4.7
Wisconsin	17.1	5.3
Wyoming	18.9	5.5

## Discussion

Across US states in 2016, prevalence of current tobacco product use varied most for cigarettes, followed by smokeless tobacco and e-cigarettes. Three states (Arkansas, Kentucky, and Tennessee) were among the top 10 states with the highest prevalence for all 3 assessed tobacco product types. As many as 1 in 4 adults were current cigarette smokers (West Virginia); 1 in 10 adults used smokeless tobacco products (Wyoming); and 1 in 15 adults currently used e-cigarettes (Oklahoma). As the tobacco product landscape continues to diversify, these findings underscore the importance of sustained monitoring and reporting of adult tobacco use across tobacco product types to help inform comprehensive tobacco use prevention and control strategies.

There has been significant public health progress in the reduction of adult cigarette smoking in the United States (1). Compared with previous state level BRFSS estimates (5), our findings suggest the prevalence of current cigarette smoking among US adults declined significantly in all 50 states and DC except Tennessee from 2011 to 2016. Nevertheless, our study reinforces that cigarette smoking is still the most common form of tobacco product used among US adults. The 2016 National Health Interview Survey estimates that 15.5% percent of adults (37.8 million) are current smokers (15). Consistent with previous observations, however, our study also indicates that cigarette smoking prevalence is higher among adults living in certain US regions, including the Midwest and the South, than in the Northeast and West (1,5,10). Thus, the statewide use of evidence-based tobacco control interventions, including tobacco product price increases, smoke-free policies, increased access to cessation services, and hard-hitting mass-media campaigns, are critical to further reduce cigarette smoking and smoking-related disease and death among US adults (1,16).

In contrast to cigarette smoking patterns, smokeless tobacco use has not declined commensurately among US adults (1). For instance, the range of smokeless tobacco use prevalence presented in this report (1.3%–9.8%) is consistent with the previously published range from 2011 (1.4%–8.9%) (5). Moreover, during 2011 through 2016, most states either experienced no significant change or an increase in the prevalence of smokeless tobacco use among adults. These trends align with the detected increase in smokeless tobacco consumption (17) and may be partly attributed to increased expenditures for advertising and promotion of these products, from $684.9 million in 2015 to $759.3 million in 2016 (18). Given that smokeless tobacco also causes substantial morbidity and premature mortality (1), these data demonstrate the public health importance of incorporating smokeless tobacco products within the framework of comprehensive tobacco use prevention and control programs.

We also presented the most recent state-specific prevalence estimates of current and ever e-cigarette use among US adults. Our findings indicate that across these jurisdictions, the prevalence of ever e-cigarette use was 3.7 (in Utah) to 7.0 (in South Dakota) times higher than the prevalence of current e-cigarette use. Although BRFSS included these indicators for the first time in 2016, the Tobacco Use Supplement to the Current Population survey assessed e-cigarette use during 2014–2015 (10). The measures used to define current and ever e-cigarette use were consistent between surveys. Variations between estimates derived from these 2 data sets may be due in part to differences in survey mode, sample size, and sampling time frame. Moreover, data from other nationally representative studies suggest that trends in ever and current e-cigarette use continue to evolve. For instance, Bao and colleagues examined data from the National Health Interview Survey between 2014 and 2016 and reported a significant increase in the prevalence of ever e-cigarette use among US adults (from 12.6% to 15.3%) but a decline in the prevalence of current e-cigarette use (from 3.7% to 3.2%) (19). These differences may be partly due to competing factors related to e-cigarette experimentation and discontinuation of their use (20).

A substantial proportion of adult tobacco users use more than 1 product (1). In 2016, current e-cigarette users were predominantly comprised of current cigarette smokers in most US states and DC. This held true even in states with low cigarette smoking prevalence. For example, although Utah had the lowest prevalence of current cigarette smoking (8.8%), it also had the highest percentage of current e-cigarette use among its adult population of current cigarette smokers (21.1%). That being said, the national prevalence of e-cigarette use has increased significantly among former and never smokers over time (19). The patterns and trajectories of tobacco product use among nonsmokers warrants further monitoring, including among adolescents, young adults, and adults more generally (21).

To our knowledge, this is the first study to assess state-specific prevalence of cigarette smoking, smokeless tobacco use, and e-cigarette use among adults in all 50 US states and DC by sex and race/ethnicity. Significant differences in state-specific tobacco product use were identified between these 2 subgroups. For instance, men had a higher prevalence of cigarette smoking than women. The use of smokeless tobacco was particularly high among men compared with women, with Wyoming having the highest prevalence of smokeless tobacco use among both men (17.0%) and women (2.3%). These differences might be partly explained by sociocultural influences, including advertising and promotion of these products, as well as varying social norms related to the acceptability of tobacco use (22,23). Furthermore, studies have demonstrated that pregnant women and women of childbearing age may be prompted to switch from cigarettes to e-cigarettes or to engage in dual use of these products (24,25). Although e-cigarettes may help nonpregnant adult smokers if used as a complete substitute for cigarettes or other combustible tobacco products, the long-term effectiveness of these products for smoking cessation is inconclusive, including for pregnant women (14). Furthermore, the prevalence of ever e-cigarette use among US adults varied widely by race/ethnicity across states. These differences are consistent with national estimates citing higher current e-cigarette use among non-Hispanic whites and non-Hispanics of other or multiple races (2). Significant differences in tobacco product use can exist by other sociodemographics characteristics, including age; for instance, the overall prevalence of current e-cigarette use is high among young adults (6,11,19). Taken together, findings from this study highlight some of the key disparities associated with use of different tobacco products, which can be minimized by comprehensive state tobacco control programs that address the diverse tobacco products being used among US adults.

The findings in this report are subject to at least 3 limitations. First, BRFSS does not include adults without wireless or landline telephone service; however, their exclusion would not be expected to introduce any major bias because only 3.1% of US adults reported having no telephone service in 2016 (26). Second, these data are self-reported and might be subject to reporting bias. Although these self-reported tobacco use estimates were not biochemically validated, previous meta-studies identified overall high concordance between self-reported tobacco product use behaviors and biochemical assessment with cotinine (27). Finally, the median state response rates ranged from 25.1% to 60.1%. Even after adjusting for nonresponse, low response rates can increase the potential for bias if there are systematic differences between respondents and nonrespondents; however, BRFSS has been shown to be valid and reliable (28).

The prevalence of adult cigarette smoking, smokeless tobacco use, and e-cigarette use varies across states. The findings have implications for interventions and programs addressing the diverse tobacco products used among US adults, including newer products such as e-cigarettes. Continued implementation of proven population-based interventions, including increasing tobacco product prices, implementing and enforcing comprehensive smoke-free laws, warning about the dangers of tobacco product use through mass media campaigns, and increasing barrier-free access to evidence-based cessation treatments such as behavioral counseling and Food and Drug Administration–approved medication, can help reduce tobacco use, particularly in states with the highest prevalence of use (16). Establishing these evidence-based strategies to encompass all tobacco products, including e-cigarettes, could minimize the potential adverse effects of e-cigarette use among vulnerable populations, including adolescents, while also maximizing any potential benefits among adult smokers who might use e-cigarettes to quit smoking completely (6,29).
